# Supplementary figures and images for: Polyphosphate nanoparticles enhance the fibrin stabilization by histones more efficiently than linear polyphosphates
Source: PLoS One. 2022 Apr 25;17(4):e0266782. doi: 10.1371/journal.pone.0266782 (PMC9037942; doi:10.1371/journal.pone.0266782)

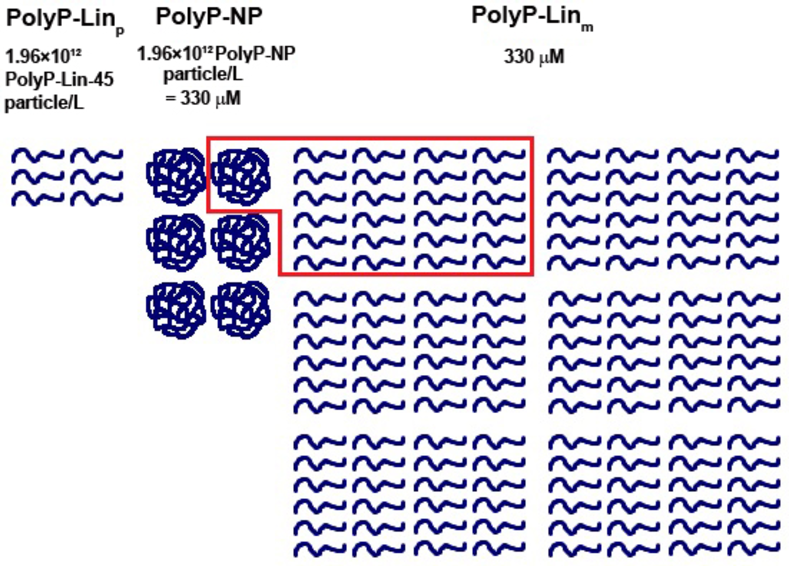

Supplement: S1 Fig — (TIF) [file pone.0266782.s001.tif]
