# Supplementary material for: Polyphosphate nanoparticles enhance the fibrin stabilization by histones more efficiently than linear polyphosphates
Source: PLoS One. 2022 Apr 25;17(4):e0266782. doi: 10.1371/journal.pone.0266782 (PMC9037942; doi:10.1371/journal.pone.0266782)
Supplement: S1 File — (ZIP) [file pone.0266782.s002.zip › PolyP-Lin_measurements_all_curves_overlay.pdf]

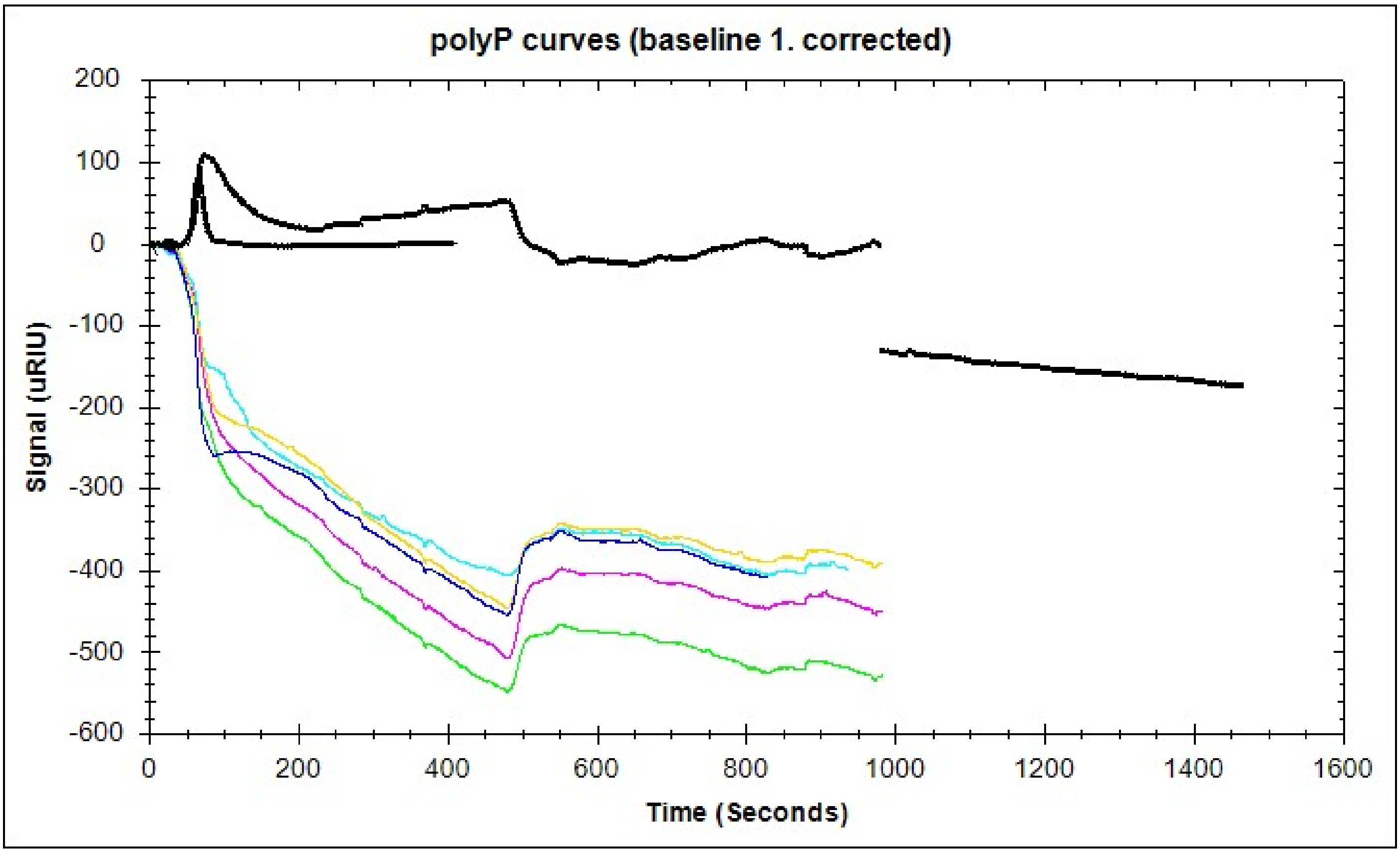

Run	Source
polyP curves	C:\Users\ABLE\Desktop\SPR_2021-05-28_elso-TraceDrawer_cikkhez_polylin.trc_original

	Curve	Ligand	Conc. (M)	Target	Source	Description
	buffer 1.		0		C:\Users\ABLE\Desktop\SPR_2021-05-28_elso-TraceDrawer_cikkhez_polylin.trc_original	
	buffer 2.		0		C:\Users\ABLE\Desktop\SPR_2021-05-28_elso-TraceDrawer_cikkhez_polylin.trc_original	
	45 polyP 1. - Reference curve		1.00e-3		C:\Users\ABLE\Desktop\SPR_2021-05-28_elso-TraceDrawer_cikkhez_polylin.trc_original	
	100 polyP 1. - Reference curve		1.00e-3		C:\Users\ABLE\Desktop\SPR_2021-05-28_elso-TraceDrawer_cikkhez_polylin.trc_original	
	100 polyP 2. - Reference curve		1.00e-3		C:\Users\ABLE\Desktop\SPR_2021-05-28_elso-TraceDrawer_cikkhez_polylin.trc_original	
	700 polyP 1. - Reference curve		1.00e-3		C:\Users\ABLE\Desktop\SPR_2021-05-28_elso-TraceDrawer_cikkhez_polylin.trc_original	
	700 polyP 2. - Reference curve		1.00e-3		C:\Users\ABLE\Desktop\SPR_2021-05-28_elso-TraceDrawer_cikkhez_polylin.trc_original	
