# Supplementary material for: Polyphosphate nanoparticles enhance the fibrin stabilization by histones more efficiently than linear polyphosphates
Source: PLoS One. 2022 Apr 25;17(4):e0266782. doi: 10.1371/journal.pone.0266782 (PMC9037942; doi:10.1371/journal.pone.0266782)
Supplement: S1 File — (ZIP) [file pone.0266782.s002.zip › PolyP-NP_kinetics_estimation.pdf]

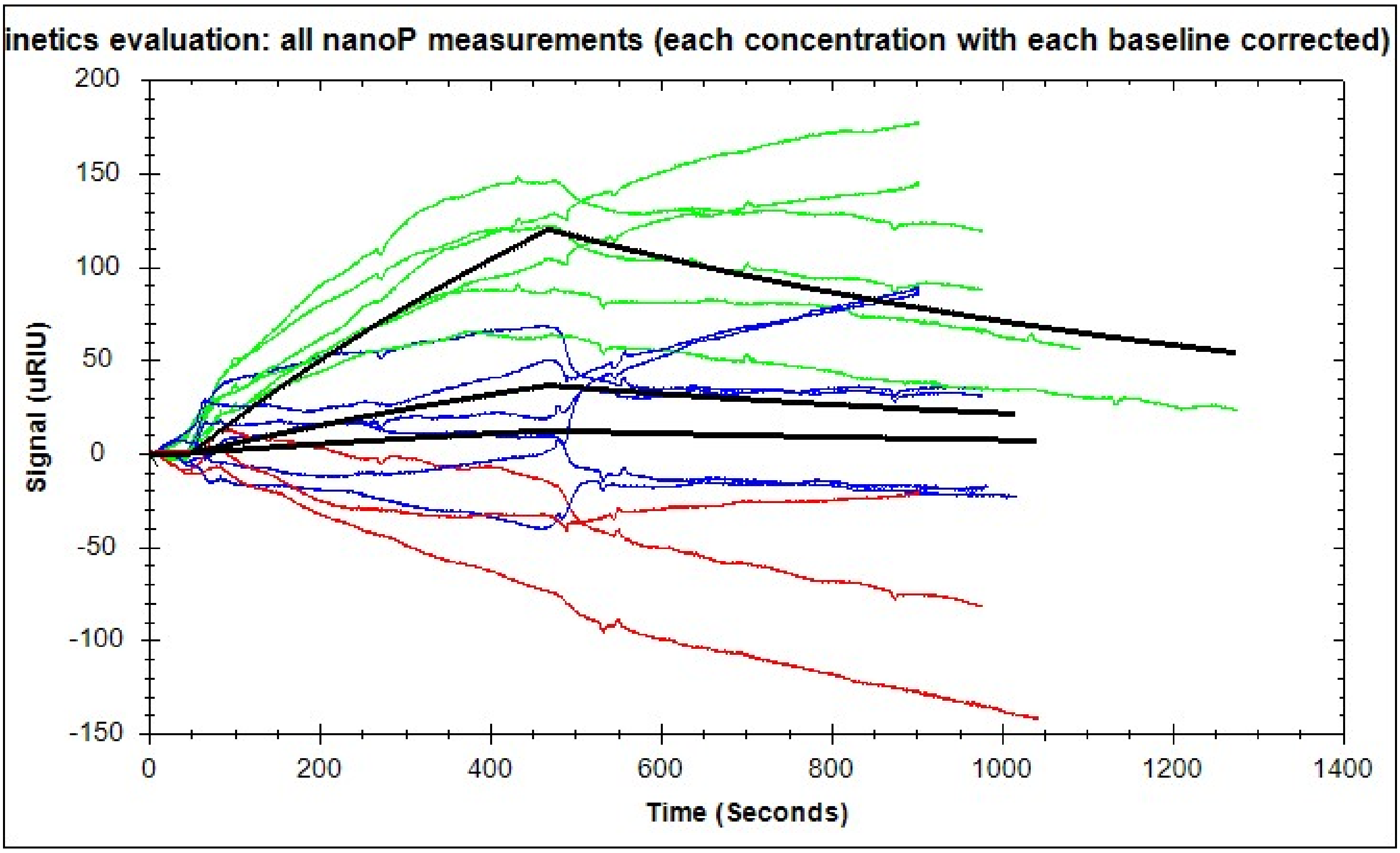

Evaluation type: OneToOne

Curve name	Bmax ([Signal (uRIU)])	ka (1/(M*s))	kd (1/s)	KD (M)	BI ([Signal (uRIU)])
1.0 nanoP 2. - Reference curve(3) fitted	3826.98	9.28e-2	9.90e-4	1.07e-2	0.00
0.3 nanoP 2. - Reference curve(3) fitted	3826.98	9.28e-2	9.90e-4	1.07e-2	0.00
1.0 nanoP 1. - Reference curve(3) fitted	3826.98	9.28e-2	9.90e-4	1.07e-2	0.00
1.0 nanoP 1. - Reference curve(2) fitted	3826.98	9.28e-2	9.90e-4	1.07e-2	0.00
0.3 nanoP 3. - Reference curve fitted	3826.98	9.28e-2	9.90e-4	1.07e-2	0.00
0.3 nanoP 3. - Reference curve(2) fitted	3826.98	9.28e-2	9.90e-4	1.07e-2	0.00
1.0 nanoP 2. - Reference curve(2) fitted	3826.98	9.28e-2	9.90e-4	1.07e-2	0.00
0.1 nanoP 1. - Reference curve(3) fitted	3826.98	9.28e-2	9.90e-4	1.07e-2	0.00
0.1 nanoP 1. - Reference curve fitted	3826.98	9.28e-2	9.90e-4	1.07e-2	0.00
0.3 nanoP 2. - Reference curve fitted	3826.98	9.28e-2	9.90e-4	1.07e-2	0.00
0.3 nanoP 2. - Reference curve(2) fitted	3826.98	9.28e-2	9.90e-4	1.07e-2	0.00
1.0 nanoP 2. - Reference curve fitted	3826.98	9.28e-2	9.90e-4	1.07e-2	0.00
1.0 nanoP 1. - Reference curve fitted	3826.98	9.28e-2	9.90e-4	1.07e-2	0.00
0.1 nanoP 1. - Reference curve(2) fitted	3826.98	9.28e-2	9.90e-4	1.07e-2	0.00
0.3 nanoP 3. - Reference curve(3) fitted	3826.98	9.28e-2	9.90e-4	1.07e-2	0.00

Curve name	Chi2 ([Signal (uRIU)]^2)	U-value: Bmax/ka (%)
1.0 nanoP 2. - Reference curve(3) fitted	1709.67	24.00
0.3 nanoP 2. - Reference curve(3) fitted	1709.67	24.00
1.0 nanoP 1. - Reference curve(3) fitted	1709.67	24.00
1.0 nanoP 1. - Reference curve(2) fitted	1709.67	24.00
0.3 nanoP 3. - Reference curve fitted	1709.67	24.00
0.3 nanoP 3. - Reference curve(2) fitted	1709.67	24.00
1.0 nanoP 2. - Reference curve(2) fitted	1709.67	24.00
0.1 nanoP 1. - Reference curve(3) fitted	1709.67	24.00
0.1 nanoP 1. - Reference curve fitted	1709.67	24.00
0.3 nanoP 2. - Reference curve fitted	1709.67	24.00

Curve name	Chi2 ([Signal (uRIU)]^2)	U-value: Bmax/ka (%)
0.3 nanoP 2. - Reference curve(2) fitted	1709.67	24.00
1.0 nanoP 2. - Reference curve fitted	1709.67	24.00
1.0 nanoP 1. - Reference curve fitted	1709.67	24.00
0.1 nanoP 1. - Reference curve(2) fitted	1709.67	24.00
0.3 nanoP 3. - Reference curve(3) fitted	1709.67	24.00

Run	Source
all nanoP measurements (each concentration with each baseline corrected)	New Overlay(14)

	Curve	Ligand	Conc. (M)	Target	Source	Description
■	0.1 nanoP 1. - Reference curve fitted		0		Kinetics evaluation.EvalItem	Kinetic fit to curve 0.1 nanoP 1. - Reference curve
■	0.3 nanoP 2. - Reference curve fitted		0		Kinetics evaluation.EvalItem	Kinetic fit to curve 0.3 nanoP 2. - Reference curve
■	0.3 nanoP 3. - Reference curve fitted		0		Kinetics evaluation.EvalItem	Kinetic fit to curve 0.3 nanoP 3. - Reference curve
■	1.0 nanoP 1. - Reference curve fitted		0		Kinetics evaluation.EvalItem	Kinetic fit to curve 1.0 nanoP 1. - Reference curve
■	1.0 nanoP 2. - Reference curve fitted		0		Kinetics evaluation.EvalItem	Kinetic fit to curve 1.0 nanoP 2. - Reference curve
■	0.1 nanoP 1. - Reference curve(2) fitted		0		Kinetics evaluation.EvalItem	Kinetic fit to curve 0.1 nanoP 1. - Reference curve(2)
■	0.3 nanoP 2. - Reference curve(2) fitted		0		Kinetics evaluation.EvalItem	Kinetic fit to curve 0.3 nanoP 2. - Reference curve(2)
■	0.3 nanoP 3. - Reference curve(2) fitted		0		Kinetics evaluation.EvalItem	Kinetic fit to curve 0.3 nanoP 3. - Reference curve(2)
■	1.0 nanoP 1. - Reference curve(2) fitted		0		Kinetics evaluation.EvalItem	Kinetic fit to curve 1.0 nanoP 1. - Reference curve(2)
■	1.0 nanoP 2. - Reference curve(2) fitted		0		Kinetics evaluation.EvalItem	Kinetic fit to curve 1.0 nanoP 2. - Reference curve(2)
■	0.1 nanoP 1. - Reference curve(3) fitted		0		Kinetics evaluation.EvalItem	Kinetic fit to curve 0.1 nanoP 1. - Reference curve(3)
■	0.3 nanoP 2. - Reference curve(3) fitted		0		Kinetics evaluation.EvalItem	Kinetic fit to curve 0.3 nanoP 2. - Reference curve(3)
■	0.3 nanoP 3. - Reference curve(3) fitted		0		Kinetics evaluation.EvalItem	Kinetic fit to curve 0.3 nanoP 3. - Reference curve(3)
■	1.0 nanoP 1. - Reference curve(3) fitted		0		Kinetics evaluation.EvalItem	Kinetic fit to curve 1.0 nanoP 1. - Reference curve(3)
■	1.0 nanoP 2. - Reference curve(3) fitted		0		Kinetics evaluation.EvalItem	Kinetic fit to curve 1.0 nanoP 2. - Reference curve(3)
■	0.1 nanoP 1. - Reference curve		1.00e-4		New Overlay(14)	
■	0.3 nanoP 2. - Reference curve		3.00e-4		New Overlay(14)	
■	0.3 nanoP 3. - Reference curve		3.00e-4		New Overlay(14)	
■	1.0 nanoP 1. - Reference curve		1.00e-3		New Overlay(14)	
■	1.0 nanoP 2. - Reference curve		1.00e-3		New Overlay(14)	
■	0.1 nanoP 1. - Reference curve(2)		1.00e-4		New Overlay(14)	
■	0.3 nanoP 2. - Reference curve(2)		3.00e-4		New Overlay(14)	
■	0.3 nanoP 3. - Reference curve(2)		3.00e-4		New Overlay(14)	
■	1.0 nanoP 1. - Reference curve(2)		1.00e-3		New Overlay(14)	
■	1.0 nanoP 2. - Reference curve(2)		1.00e-3		New Overlay(14)	
■	0.1 nanoP 1. - Reference curve(3)		1.00e-4		New Overlay(14)	
■	0.3 nanoP 2. - Reference curve(3)		3.00e-4		New Overlay(14)	
■	0.3 nanoP 3. - Reference curve(3)		3.00e-4		New Overlay(14)	
■	1.0 nanoP 1. - Reference curve(3)		1.00e-3		New Overlay(14)	
■	1.0 nanoP 2. - Reference curve(3)		1.00e-3		New Overlay(14)	
