# Supplementary material for: Polyphosphate nanoparticles enhance the fibrin stabilization by histones more efficiently than linear polyphosphates
Source: PLoS One. 2022 Apr 25;17(4):e0266782. doi: 10.1371/journal.pone.0266782 (PMC9037942; doi:10.1371/journal.pone.0266782)
Supplement: S1 File — (ZIP) [file pone.0266782.s002.zip › PolyP-NP_measurements_all_curves_overlay.pdf]

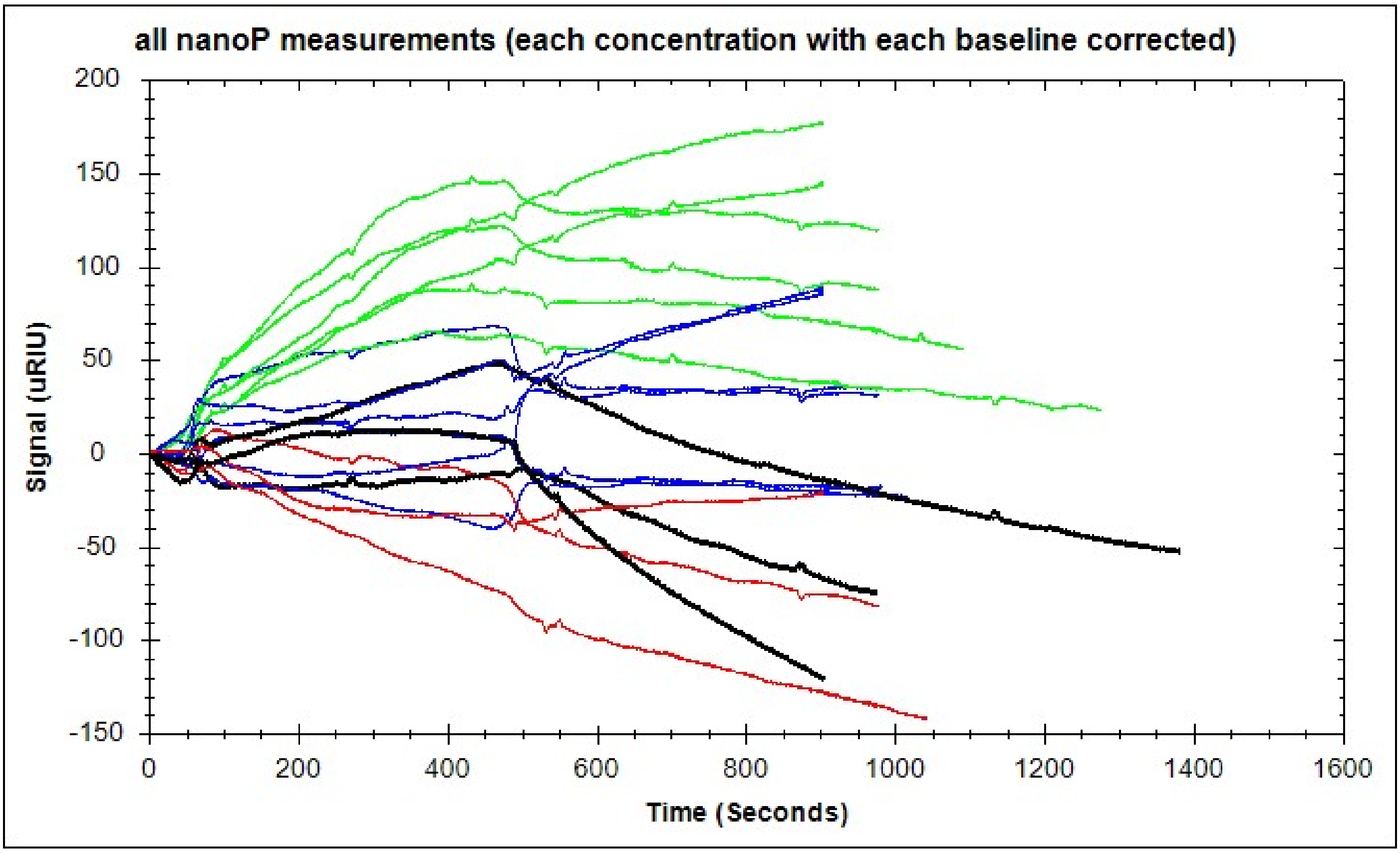

Run	Source
nanoP curves (baseline 1. corrected)	New Overlay(11)
nanoP curves (baseline 2. corrected)	New Overlay(12)
nanoP curves (baseline 3. corrected)	New Overlay(13)

	Curve	Ligand	Conc. (M)	Target	Source	Description
	buffer 1. (nanoP)		0		New Overlay(11)	
	0.1 nanoP 1. - Reference curve		1.00e-4		New Overlay(11)	
	0.3 nanoP 2. - Reference curve		3.00e-4		New Overlay(11)	
	0.3 nanoP 3. - Reference curve		3.00e-4		New Overlay(11)	
	1.0 nanoP 1. - Reference curve		1.00e-3		New Overlay(11)	
	1.0 nanoP 2. - Reference curve		1.00e-3		New Overlay(11)	
	buffer 2. (nanoP)		0		New Overlay(12)	
	0.1 nanoP 1. - Reference curve(2)		1.00e-4		New Overlay(12)	
	0.3 nanoP 2. - Reference curve(2)		3.00e-4		New Overlay(12)	
	0.3 nanoP 3. - Reference curve(2)		3.00e-4		New Overlay(12)	
	1.0 nanoP 1. - Reference curve(2)		1.00e-3		New Overlay(12)	
	1.0 nanoP 2. - Reference curve(2)		1.00e-3		New Overlay(12)	
	buffer 3. (nanoP)		0		New Overlay(13)	
	0.1 nanoP 1. - Reference curve(3)		1.00e-4		New Overlay(13)	
	0.3 nanoP 2. - Reference curve(3)		3.00e-4		New Overlay(13)	
	0.3 nanoP 3. - Reference curve(3)		3.00e-4		New Overlay(13)	
	1.0 nanoP 1. - Reference curve(3)		1.00e-3		New Overlay(13)	
	1.0 nanoP 2. - Reference curve(3)		1.00e-3		New Overlay(13)	
